# Supplementary material for: Mapping nanocrystalline disorder within an amorphous metal–organic framework
Source: Commun Chem. 2023 May 11;6:92. doi: 10.1038/s42004-023-00891-9 (PMC10175482; doi:10.1038/s42004-023-00891-9)
Supplement: Supplementary file 2 — Supplementary information [file 42004_2023_891_MOESM2_ESM.pdf]

Mapping Nanocrystalline Disorder Within an Amorphous Metal–Organic Framework

SUPPLEMENTARY INFORMATION

Adam F. Sapnik,[1] Chao Sun,[2] Joonatan E. M. Laulainen,[1] Duncan N. Johnstone,[1] Rik Brydson,[2] Timothy Johnson,[3] Paul A. Midgley,[1] Thomas D. Bennett[1] and Sean M. Collins.[1,2,4]*

1. Department of Materials Science and Metallurgy, University of Cambridge, Cambridge, UK.

2. School of Chemical and Process Engineering, University of Leeds, Leeds, UK.

3. Johnson Matthey Technology Centre, Blount's Court, Sonning Common, Reading, UK.

4. School of Chemistry, University of Leeds, Leeds, UK.

Email: S.M.Collins@leeds.ac.uk

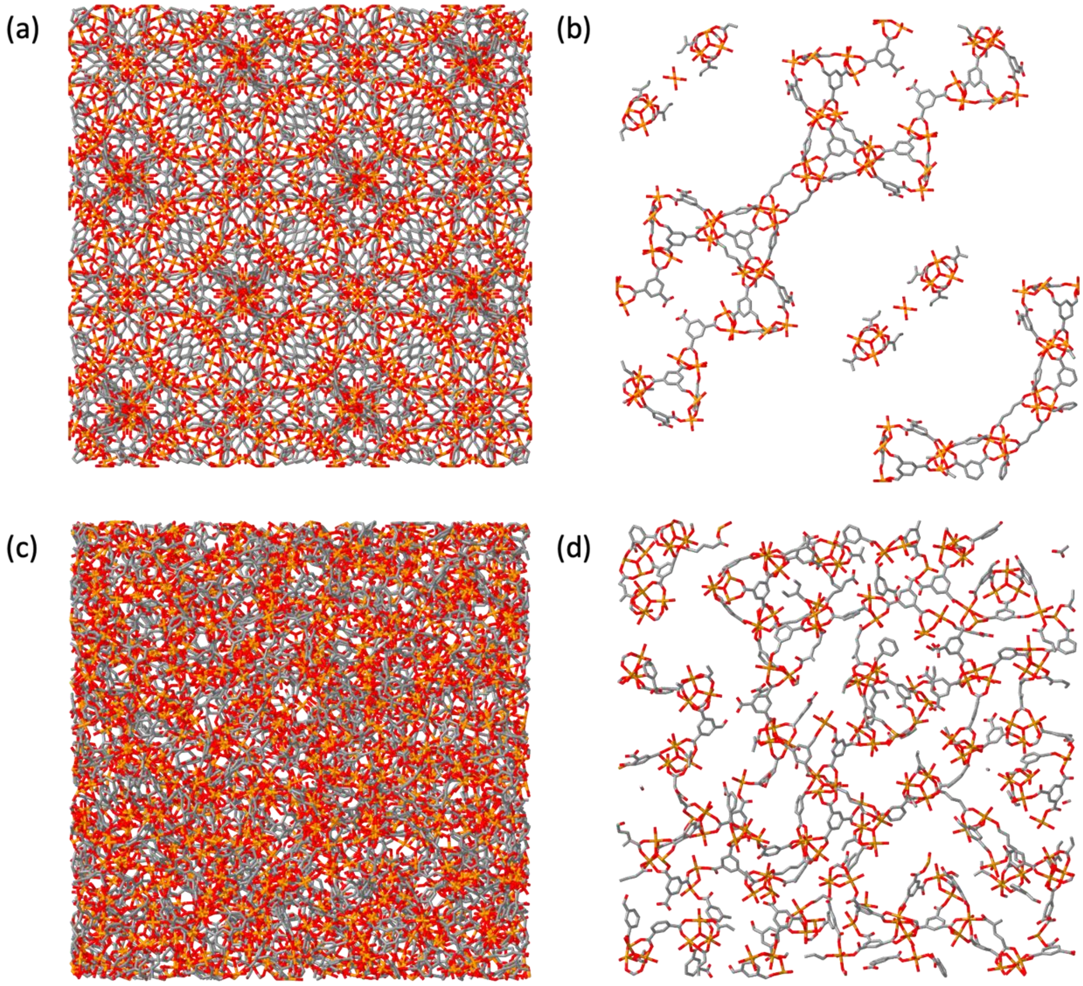

Supplementary Figure 1. (a) Crystallographic unit cell for MIL-100, viewed down the *a* axis. (b) A 10 Å slice of the MIL-100 unit cell. (c) A representative model (~80 Å in length) of the average amorphous matrix in Fe-BTC. (d) A 10 Å slice of the Fe-BTC model. Fe (orange), O (red), C (grey) and H omitted for clarity.

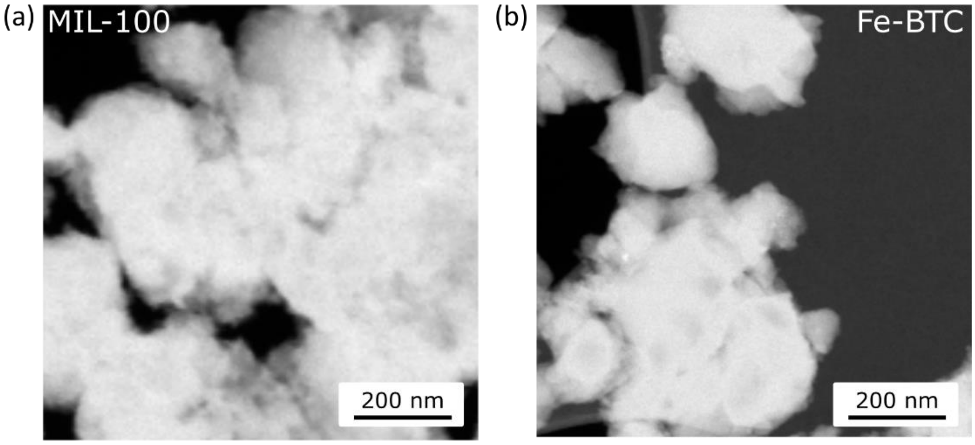

Supplementary Figure 2. Overview ADF-STEM micrographs for (a) MIL-100 and (b) Fe-BTC particles prepared on a lacey carbon support film.

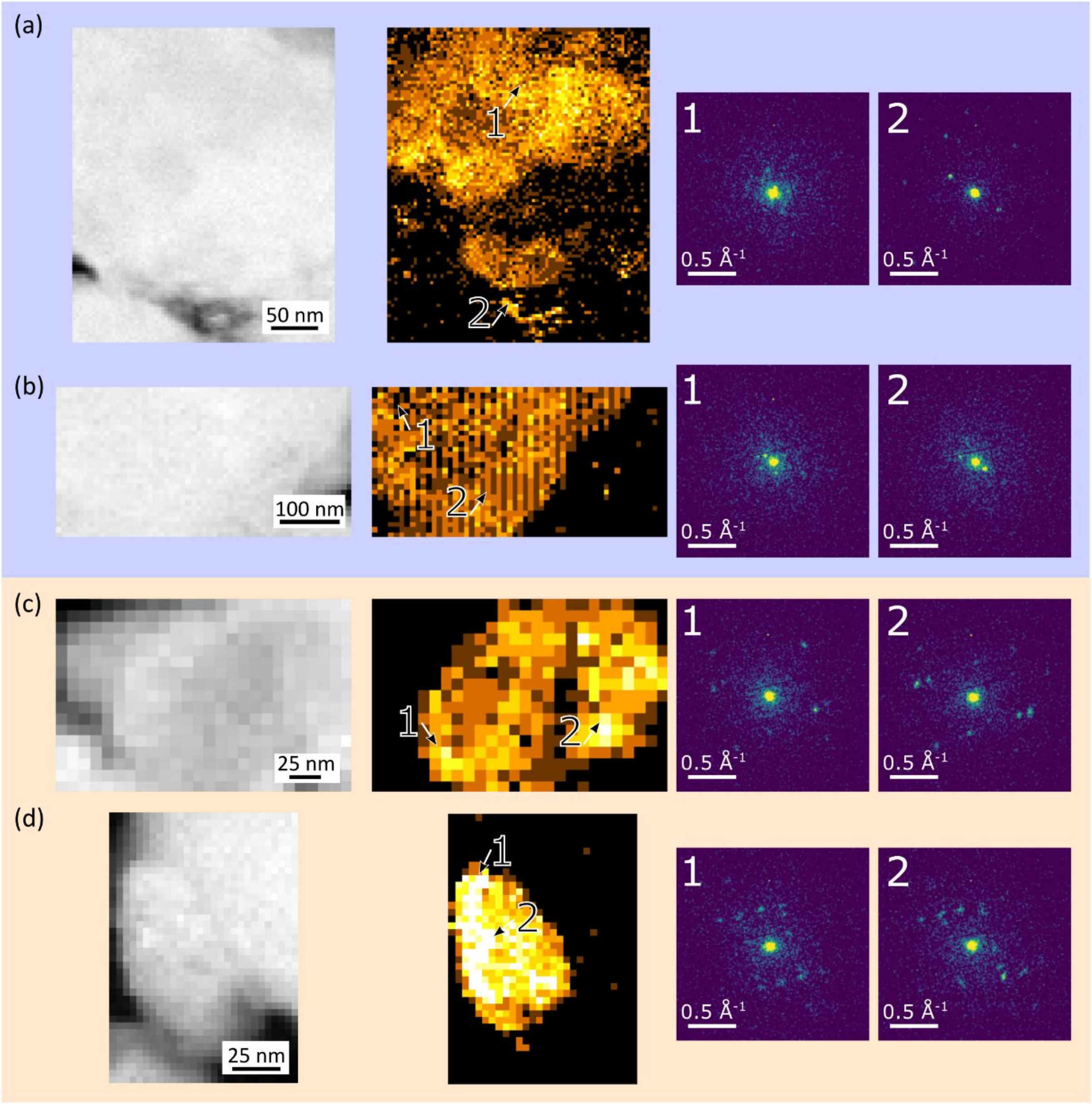

Supplementary Figure 3. ADF-STEM (grayscale), crystallinity maps (amber), and single-pixel diffraction patterns (Viridis colourmap) for additional examples of (a)-(b) MIL-100 and (c)-(d) Fe-BTC crystalline domains. Arrows mark the positions of the numbered single-pixel patterns. Diffraction patterns are plotted as the square root of the recorded intensity.

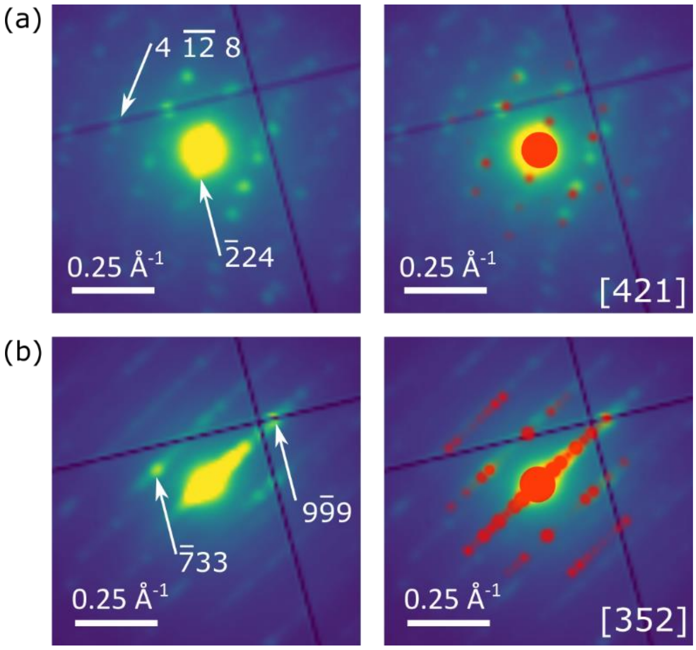

Supplementary Figure 4. Area-averaged electron diffraction patterns acquired by SED from MIL-100 crystals shown in (a) Fig. 6b and (b) Fig. 3b. The overlayed simulated diffraction patterns (red) show close correspondence for the principal points. Some additional orientations contribute as a result of area-averaging, giving rise to some additional Bragg spots that show the same periodicities and are consistent with small rotations or tilts (mosaicity) in this large unit cell structure.

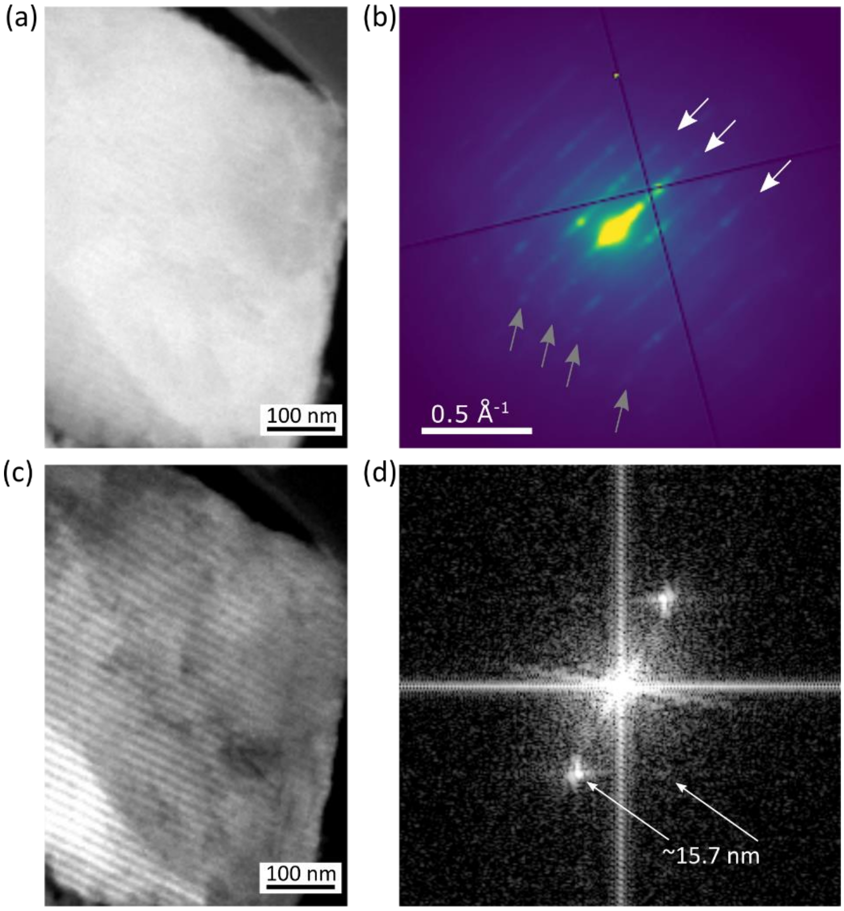

Supplementary Figure 5. (a) ADF-STEM image (inner angle, outer angle) and (b) corresponding diffraction pattern averaged over the field of view. Grey arrows mark where reflections indicate possible contributions from a second orientations with a slight out-of-plane rotation applied relative to the rows of reflections marked with white arrows. The diffraction pattern is plotted as the square root of the recorded intensity. (c) Fringe pattern observed in the low-angle ADF-STEM (inner angle, outer angle) micrograph showing signature is a coherent, diffraction contrast effect with (d) corresponding Fourier transform of the fringe image showing spacings of approximately 15.7 nm, consistent with a moiré fringe between two identical lattices with a small rotation between them.

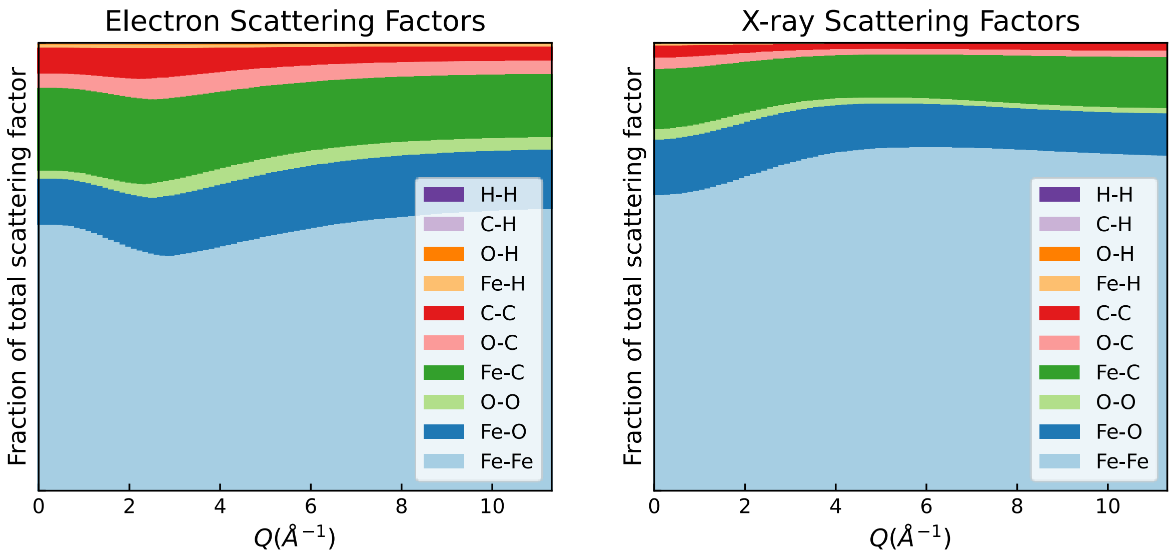

Supplementary Figure 6. *Q*-dependent relative scattering factors for each pair-pair partial scattering factor, as a fraction of the total scattering factor, for electrons (left) and X-rays (right). The top of each figure is 1 and represents the sum of all pair-pair partial scattering factors. Both X-ray and electron scattering factors are dominated by Fe-based pairs, particularly Fe-Fe scattering. Scattering from C and O based-pairs (such as C-C and O-C in red) contribute substantially less in X-ray scattering than in electron scattering. Additionally, the *Q*-dependence varies between X-ray and electron scattering.

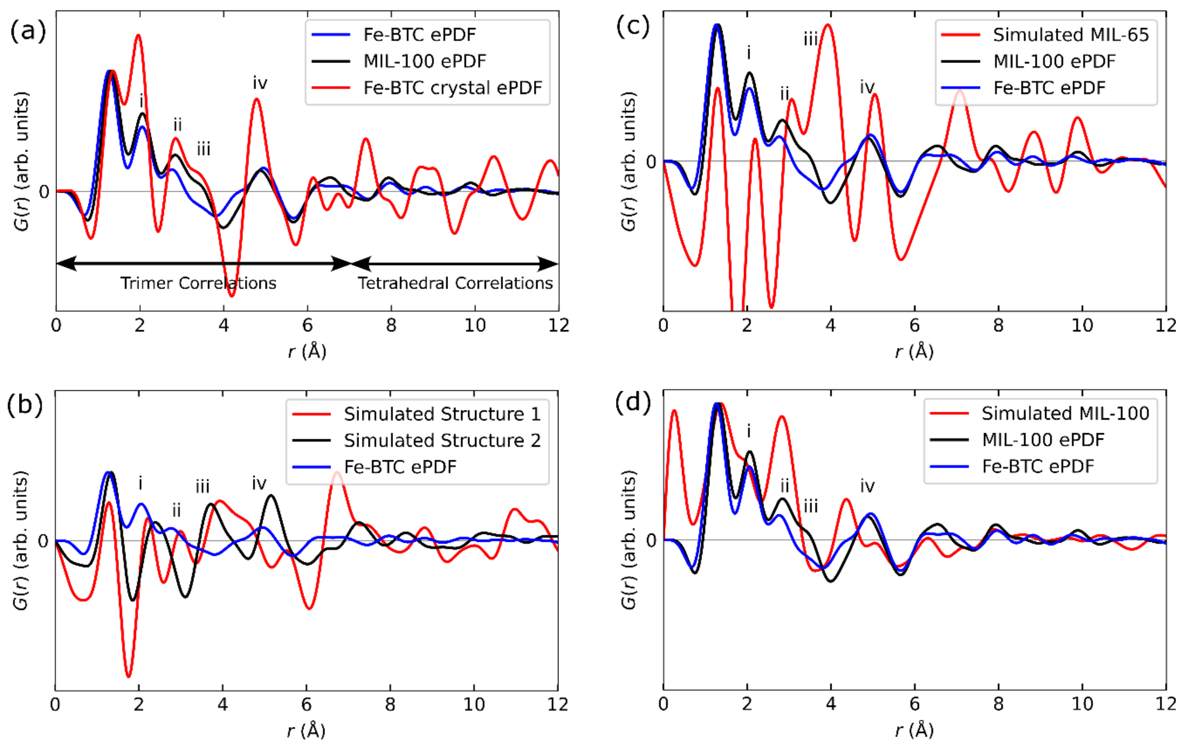

Supplementary Figure 7. Additional acquired and simulated ePDFs. (a) Acquired amorphous Fe-BTC ePDF (blue) and average MIL-100 (black) compared to an ePDF acquired by summing crystalline domains observed in Fe-BTC (red). Each domain was normalised in intensity before summing. The orientational average is likely not complete for ePDFs of crystalline fractions. (b) Acquired Fe-BTC ePDF (blue) compared with two simulated crystal structures (Structure 1: 171986 CCDC, Structure 2: 171987 CCDC)[1] that show similar crystalline peak positions to the observed crystalline domains. The ePDFs are not consistent. (c) and (d) Acquired Fe-BTC ePDF (blue) and MIL-100 (black) compared to simulated MIL-65 (c) and MIL-100 (d) ePDFs. The MIL-65 structure is based on a dimer rather than a trimer structure. Peak positions are not consistent, especially compared to MIL-100 (d). The reported MIL-100 unit cell was used without further modification. H atoms were not included, which may contribute to reduced consistency between the simulated and observed MIL-100 ePDFs.

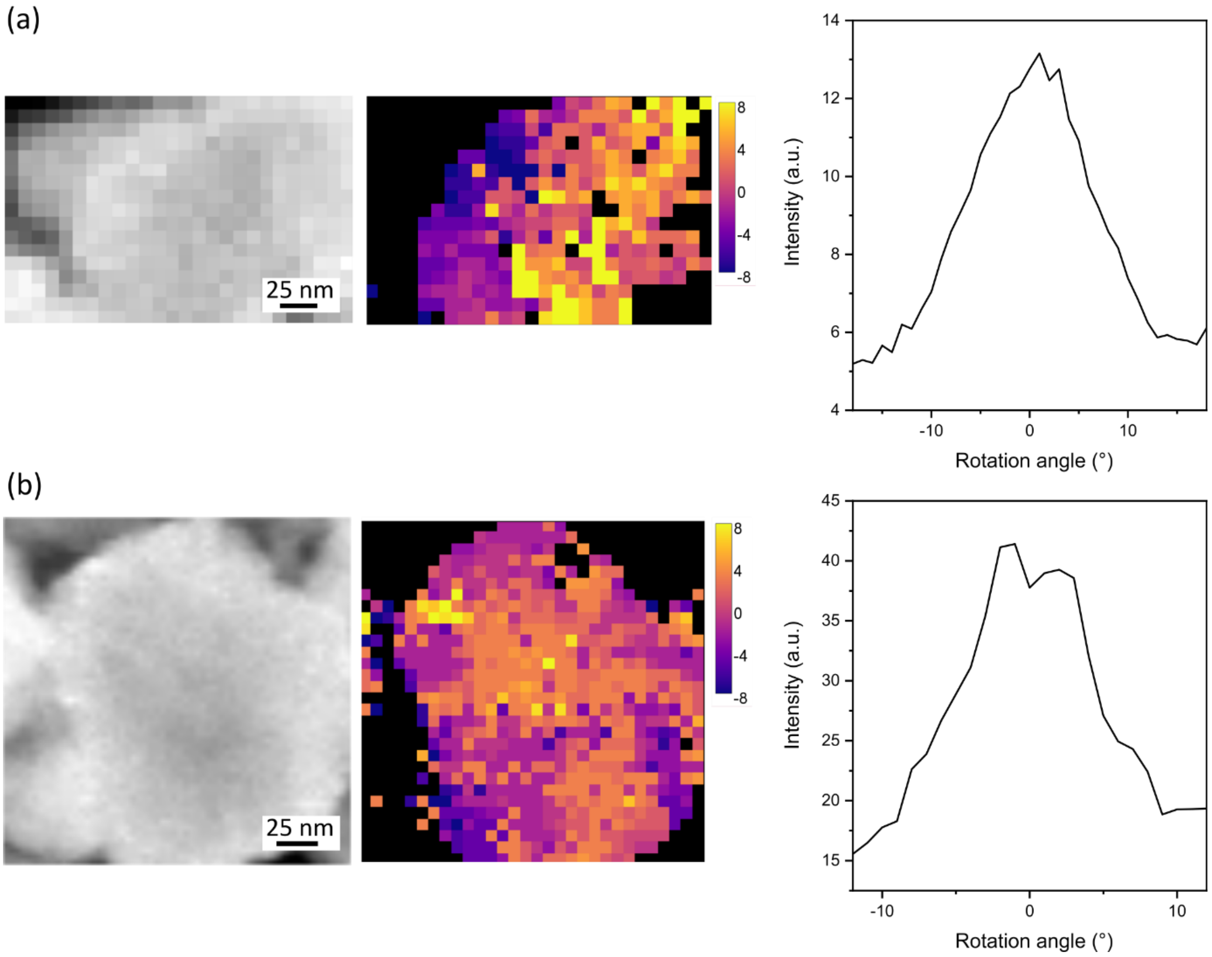

Supplementary Figure 8. Additional examples of (a), (b) two additional exhibiting continuous rotational variation in Fe-BTC. An ADF-STEM micrograph (grayscale) is shown with a corresponding map of the local angular orientation as determined from a series of Friedel pair virtual dark field images. The integrated virtual dark field intensity is plotted on the right for each case showing a peak with a full width at half-maximum of 10–15°.

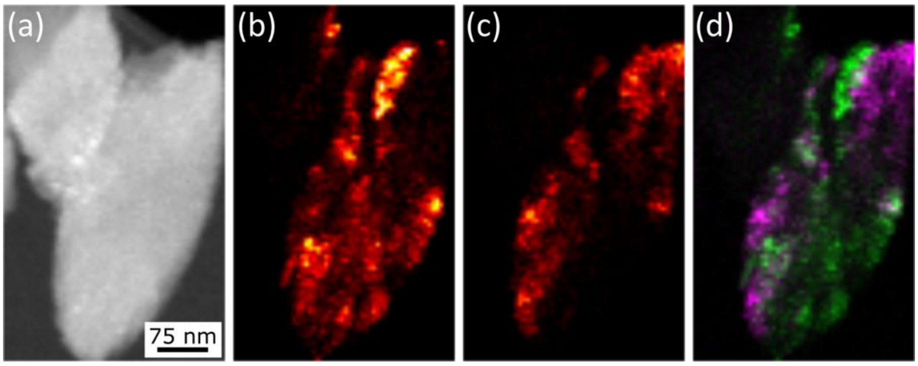

Supplementary Figure 9. (a) ADF-STEM micrograph of an Fe-BTC crystalline domain showing rotational disorder. Virtual dark fields for (b) −3° and (c) +3° and (d) colour overlay of the two, highlighting approximately diagonal arrangement of the rotationally offset regions of the crystalline domain.

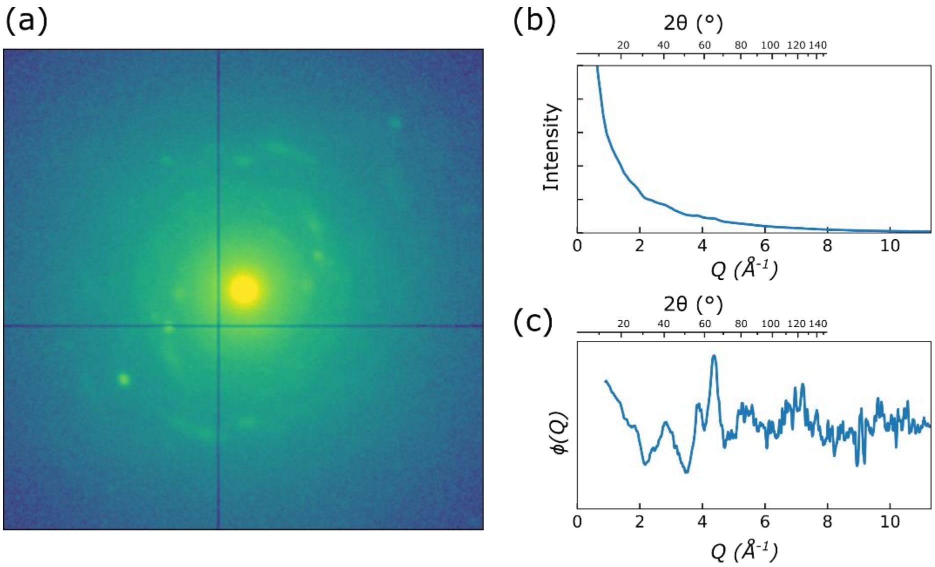

Supplementary Figure 10. (a) Summed diffraction patterns of the crystalline Fe-BTC fraction. This pattern, effectively an average pattern, exhibits strong 'spottiness' indicative of poor sampling. (b) The corresponding azimuthal profile, acquired by integration around the direct beam. The cross present due to gaps in the detector was removed prior to azimuthal integration. (c) The profile with unstructured scattering subtracted, the ϕ(Q) input used for ePDF calculation. The upper horizontal axes show the corresponding 2θ range (Cu Kα1 = 1.5406 Å).

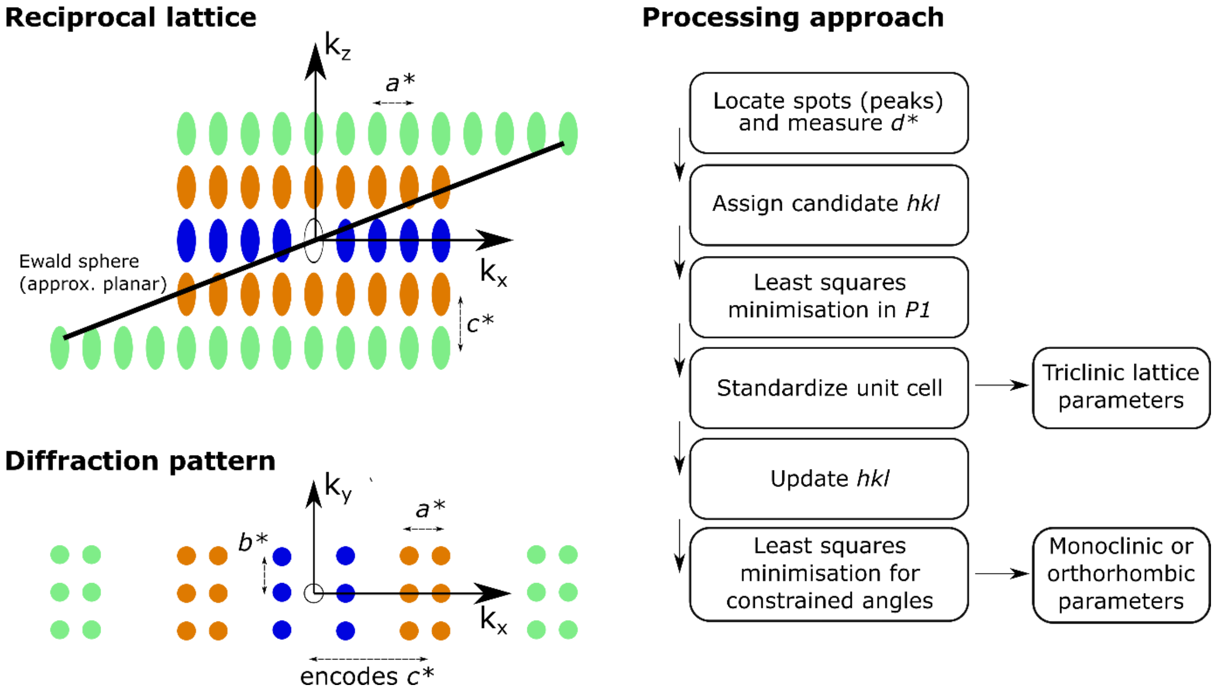

Supplementary Figure 11. (Left) Schematic overview of the approach used for the determination of lattice parameters. The reciprocal lattice is shown for an *xz* plane cut through the three-dimensional reciprocal lattice, with reciprocal lattice points elongated due to the use of thin samples along the beam trajectory. The corresponding diffraction pattern is approximately an *xy* plane cut through the reciprocal lattice by a nearly planar Ewald sphere (short electron wavelength). (Right) The processing approach is also outlined in sequential steps used to produce and refine lattice parameter estimates.

Supplementary Table 1. Lattice parameters for two diffraction patterns from Fe-BTC, labelled Fe-BTC ***1*** and Fe-BTC ***2***. Constrained refinements are denoted with an additional ***a, b, c***. Lattice parameters and parameter-specific variances were determined using the curve_fit function in SciPy. All estimated uncertainties are given as one standard deviation. The monoclinic parameters are given in non-standard form for easy comparison with the triclinic and orthorhombic parameters.

<table>
<tr><th></th><th>Fe-BTC 1a
(Triclinic)</th><th>Fe-BTC 1b
(Monoclinic)</th><th>Fe-BTC 1c
(Orthorhombic)</th><th>Fe-BTC 2a
(Triclinic)</th><th>Fe-BTC 2b
(Orthorhombic)</th></tr>
<tr><td>a</td><td>2.55 ± 0.69 Å</td><td>2.50 ± 0.09 Å</td><td>2.20 ± 0.032 Å</td><td>2.90 ± 0.023 Å</td><td>2.90 ± 0.02 Å</td></tr>
<tr><td>b</td><td>7.14 ± 0.28 Å</td><td>7.10 ± 0.06 Å</td><td>7.40 ± 0.050 Å</td><td>7.16 ± 0.24 Å</td><td>7.42 ± 0.25 Å</td></tr>
<tr><td>c</td><td>7.67 ± 1.1</td><td>7.64 ± 0.14 Å</td><td>8.27 ± 0.14 Å</td><td>10.64 ± 2.1 Å</td><td>10.49 ± 2.1</td></tr>
<tr><td>α</td><td>96.9 ± 9.7°</td><td>97.8 ± 1.5°</td><td rowspan="3">90°</td><td>90.4 ± 2.1°</td><td rowspan="3">90°</td></tr>
<tr><td>β</td><td>91.2 ± 21°</td><td rowspan="2">90°</td><td>93.9 ± 7.9°</td></tr>
<tr><td>γ</td><td>91.6 ± 11°</td><td>92.5 ± 3.2°</td></tr>
</table>

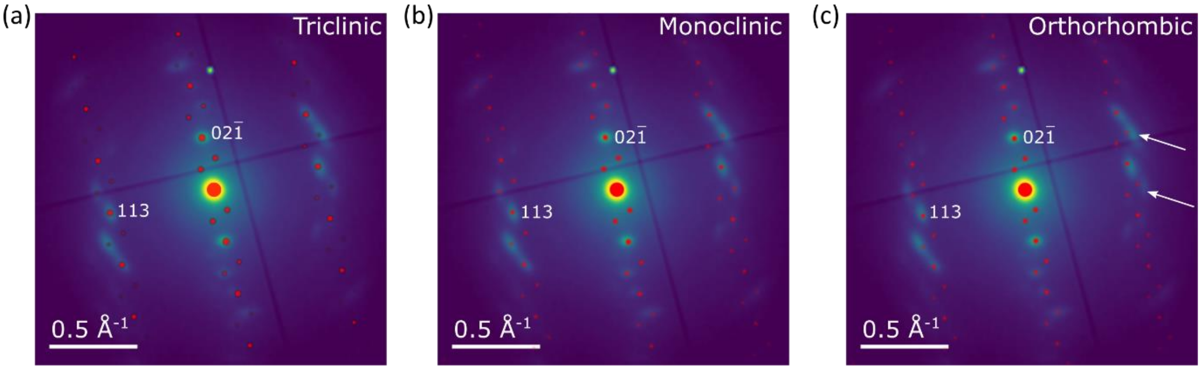

Supplementary Figure 12. Overlays showing experimental diffraction data and kinematical simulations of electron diffraction for (a) triclinic, (b) monoclinic, and (c) orthorhombic crystal systems after least squares minimisation to identify lattice parameters for Fe-BTC ***1***. The constrained fitting reduces apparent uncertainties (Supplementary Table 1), but the orthorhombic constraints for Fe-BTC ***1*** appear to reduce the quality of the fit with experimental data. White arrows highlight points in the orthorhombic-constrained unit cell that show inferior agreement with experimental data.

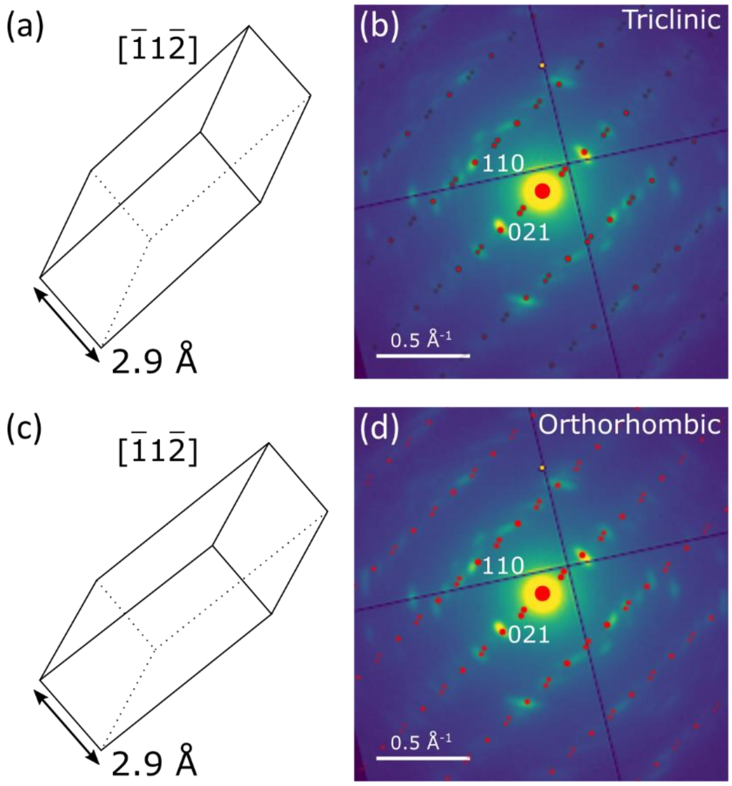

Supplementary Figure 13. (a) Second candidate unit cell for Fe-BTC (Fe-BTC ***2***) determined from the positions of Bragg peaks in (b) the corresponding diffraction pattern (see also **Fig. 7b**), viewed along the $[\bar{1}1\bar{2}]$ direction. In (a)-(b) the lattice parameters were determined with no constraints in the triclinic crystal systems (Fe-BTC ***2a***). (c) Candidate unit cell for Fe-BTC ***2*** with orthorhombic crystal system constraints applied with (d) an overlay of simulated and experimental diffraction patterns showing a comparable match with fewer free parameters. The peak positions for the unit cell are overlaid in red on the experimental diffraction pattern, with experimental intensities plotted as the square root of recorded intensities to simultaneously visualise high and low-intensity features.
